# Telerehabilitation: Development, Application, and Need for Increased Usage in the COVID-19 Era for Patients with Spinal Pathology

**DOI:** 10.7759/cureus.10563

**Published:** 2020-09-21

**Authors:** Brian Fiani, Imran Siddiqi, Sharon C Lee, Lovepreet Dhillon

**Affiliations:** 1 Neurosurgery, Desert Regional Medical Center, Palm Springs, USA; 2 College of Osteopathic Medicine, Western University of Health Sciences, Pomona, USA

**Keywords:** telerehabilitation, telerehab, physical therapy, covid, spine, spine rehab, spinal cord injury, innovation, physiotherapy, neurorehab

## Abstract

The coronavirus disease 2019 (COVID-19) pandemic has triggered governments worldwide to implement severe restrictions on physical therapy protocols in order to better control the spread of the virus. One of the mechanisms of providing physical therapy patient care during this era is via telemedicine. Telerehabilitation or telerehab is a technological visual-audio system that serves patients, including those with a spine injury, ailment, or postoperatively, with neurological deficits. In this scoping review, we discuss the development of telerehab, the technological advances in the field, and the usage of telerehab specifically pertaining to spine patients, and comment on the advancement of telerehab in the time of COVID-19. There is preliminary evidence that suggests that the adoption of telerehab in lieu of face-to-face interventions is beneficial for reducing pain and improving physical function in patients afflicted with chronic nonmalignant musculoskeletal pain from low back pain, lumbar stenosis, neck pain, and osteoarthritis. Availability is important, as the necessary technology should be accessible to all participants. Safety and security should be addressed, as the passage of patient data over the Internet requires secure confidentiality. Ease-of-use is crucial to promote practicality, user-friendly operation, and adherence to therapy. The combination of evidence-based methodologies with cost-effective services will serve as a basis for the further expansion of vital telerehab services and increases reimbursement by health insurance providers.

## Introduction and background

The coronavirus disease 2019 (COVID-19) pandemic has triggered governments worldwide to implement severe restrictions, such as social distancing, mandatory quarantines, and school closures, in an attempt to better control the spread of the virus [1]. Additionally, in order to mitigate the burden on health care systems, resources have been redirected toward high acuity levels of care and those afflicted with COVID-19. Although this shift in the healthcare landscape has been a necessary step in the fight against this deadly virus, it has certainly placed significant barriers for medical professionals who serve patients in less urgent care settings such as spine-focused physical therapy [2]. Due to the COVID-19 crisis, medical professionals have increased the usage of telemedicine, in which medical information is distributed via electronic means between individuals who are in different locations [2]. In response to this new landscape of medical practice, the World Confederation for Physical Therapy published a position statement on the use of telerehabilitation, or telerehab, in order to improve accessibility to care, and offered physical therapists an opportunity to deliver care in accordance with a new model of delivery [2]. Other national organizations, such as the American Physical Therapy Association, Australian Physiotherapy Association, and Italian Physiotherapy Association, have expanded resources and advice for the implementation of telerehab services. In stroke patients, the literature supports the utilization of telemedicine-based rehabilitation as compared to traditional face-to-face therapy [3].

Additionally, there is preliminary evidence that suggests that the adoption of telerehab in lieu of face-to-face interventions is beneficial for reducing pain and improving physical function in patients afflicted with chronic nonmalignant musculoskeletal pain from low back pain, lumbar stenosis, neck pain, and osteoarthritis [4]. Telerehab interventions have proven to be beneficial for patients suffering from low back pain to reduce pain levels and maintain that improvement via booster sessions delivered through a mobile phone application and videoconferencing [5-6]. With the unique circumstances of the COVID-19 era, telerehab would allow for the delivery of rehabilitation interactions on a larger scale [7]. The next logical step in rehabilitation is the adoption of the telemedicine model, especially pertaining to those requiring neurorehabilitation, but this is certainly not without unique challenges. Since March 2020, the Centers for Medicare Services will reimburse therapists for certain telerehab services secondary to the COVID-19 crisis. Prior to the COVID-19 era, therapists were not allowed to bill insurance companies for telemedicine services [8]. Many patients who would require telerehab care would be at a higher risk for exposure if they had to be present in a clinic for therapy. This is not to say that the telerehab system would work for all patients, such as those with limited access to the Internet, those who are unable to acquire the necessary equipment, or patients without any support at home. Regardless, there is still a large population of patients with neurologic rehabilitation requirements, and the current practice environment represents an opportunity to meet this demand with creative telehealth solutions. Billing and reimbursement for telerehab will continue to evolve, and despite the Medicare update of e-visits, it is possible that this coverage could be reversed once the COVID-19 pandemic is better controlled.

In the last decade, the technology for remote assessment, as well as intervention, in rehabilitation has vastly grown, enabling the development of telerehab. The typical services included in a physical therapy telerehab visit would include evaluation, assessment, intervention, monitoring, education, and coaching. The technologies utilized to facilitate telerehab include various forms of communication, including telephone, messaging, email, multimodal systems like videoconferencing, web-based platforms, and virtual reality [9]. In this contemporary review, we discuss the development of telerehab, the technological advances in the field, and the usage of telerehab specifically pertaining to spine patients, and comment on the advancement of telerehab in the time of COVID-19.

## Review

Technology and technical features

The technology utilized in telerehab is ideally selected with the patient in mind. The basic and fundamental principles of the physical rehabilitation process, including the evaluation of a dysfunction, planning with evidence-based principles, delivery of the selected intervention, adaption of biosystems in response to the input, and cyclical employment of the cycle to improve the patient’s condition over the course of rehabilitation [10]. Keeping those principles in mind, telerehab presents new opportunities and challenges. The first step of telerehab is remote assessment. With an accurate patient assessment, it is possible to establish a functional baseline for the patient, evaluate the efficacy of the treatment, and integrate data in order to gauge progression [11]. The basic component of every assessment is the patient interview, which can be completed via the remote assessment. Additionally, a thorough physical exam, including observation, movement exams, standardized tests, specific testing, and range of motion, may be done with some creative adaptations and utilization of technology. The observation of a patient's functionality and gait is relatively straightforward with videoconferencing technology [12]. Palpation, on the other hand, cannot be done in traditional fashion; instead, the patient may be asked to participate in self palpation with real-time demonstration and verbal guidance from the clinician. Movement exams are another challenge with the lack of clinic-based measurement tools; however, specialized rehabilitation tools, such as the “eHAB” videoconferencing technology, have been designed, which integrate videoconferencing, measurement, media tools, and assessment protocols [13]. Specific testing of muscles, nerves, or articulation would present another challenge but can be circumnavigated utilizing home-based caregivers, family members, or the patient to act as hands for the clinician, carrying out modified neurological tests, which can provide crucial information to the clinician. Other testing, such as range of motion, may be done dynamically in real-time, utilizing optics to detect joints and generating a “point cloud” of the patient, enabling accurate anthropometric measurements [14]. Motion sensors and body monitoring technology have been developed [15]. Motion sensors are devices that contain a physical mechanism or electronic sensor that quantifies motion and may be integrated with other devices. Examples include accelerometers for determining position in space, as well as the rate of movement [15]. Physiologic monitoring sensors are also available for quantifying blood pressure, body temperature, conducting an electrocardiogram, and may have contactless sensors for an electromyogram [9].

One way to categorize telerehab technology is on the basis of asynchronous versus synchronous, or real-time, communication [9]. With asynchronous or “store and forward” technology, information may be recorded, transferred, and stored either locally or on a server [16]. By the nature of this technology, there is a delay between when data is sent and when it is received. Such examples include secure messaging, email, or web-based applications. Recent technology that falls under the “store and forward” model includes virtual reality and wearable devices, which send data to the therapist. An advantage of this model of technology is that viewing and commenting on data can be done at the convenience of the patient and provider and is also less dependent on Internet connectivity. Synchronous telehealth technology, on the other hand, is done in real-time, with data being transferred in a live format. The limitation of this technology is the need for higher bandwidth, investment in hardware, and constant connectivity [17].

Another way to describe telerehab technologies is by the sensory modality utilized. One of the simplest and most cost-effective technologies utilized is text-based. Some common examples include email exchange and cell phone-messaging to transmit language-based information such as professional education and therapeutic communication. An example of non-language text-based communication is the transmission of acceleration data from a wheelchair that can be remotely emailed to a clinician evaluating the patient's overall fitness and activity level over time [18].

Audio-based technologies enable the transmission of audio data from one location to another. Plain old telephone service (POTS) technologies are widely available in most US households, are easy to use, and have low implementation costs [9]. However, there has been significant advancement beyond traditional phone lines, although the usage of the telephone is the most common audio technology utilized for telerehab. Additionally, the storage of audio files on phones, iPads, or mp3 players can be replayed, ranging from lectures on rehab topics to reviews of instruction on assistive technology. Vision-based technology includes webcams for video conferencing and video hosting platforms for synchronous communication, requiring significant bandwidth and security for transmitted images.

One exciting new domain in the telerehab field is virtual reality (VR). VR enables the patient to be presented with a multitude of visual, auditory, tactile/haptic, or even olfactory sensations as a representation of physical experience [18]. Additionally, there has been the development of haptic systems that include tactile information, referred to as force feedback in applications. Haptic technology interfaces with a patient through a sense of touch by applying force, vibration, or motion [19]. This allows further immersion in the virtual environment. One emerging field within haptic technology is the development of rehab robotics. This technology leverages traditional therapy and is intended for patients with motor disabilities, with the goal of improving motor function, shortening rehab times, and providing objective parameters for patient evaluation [20]. One advantage of utilizing VR is that it avoids the unnecessary risks that may come with performing that same task in a real or dangerous environment. Additionally, the environment can be tailored relative to the patient's condition and optimize the difficulty within the environment in accordance with the neurological severity of the particular patient [21]. A new trend within the telerehab sector is the development of applications through multimedia and gamification techniques in the rehabilitation of patients with physical disabilities. One example is “Rehab@Home,” which is an infrastructure based on commercially available and relatively low-cost devices, such as “Kinect” or “Nintendo Wii,” creating a virtual environment for the patient, and uses “exergames” that increase the engagement of rehabilitation [22]. Additionally, OPTI-RERC - Optimizing Participation Through Technology for Successful Aging with Disability - is a project that is providing VR and gamification in the home-based motor assessment. It also makes use of commercially available technologies like the “Sony Eyetoy” (Sony Corporation, Tokyo, Japan) [23]. This platform, in particular, analyzes the movement of upper and lower limbs, as well as balance impairment [23]. Other examples include “AMMR” mixed reality rehabilitation, and “Mercury,” a wearable sensor network platform for high-fidelity motion analysis [24].

When choosing a particular telerehab technology, the clinician must consider several key factors. Availability is important, as the necessary technology should be accessible to all participants. Safety and security should be addressed, as the passage of patient data over the Internet requires secured confidentiality. Ease-of-use is crucial to promote practicality, user-friendly operation, and adherence to therapy. Other factors that should be considered are medico-legal, cost of use, and interoperability with other technologies. With regards to platform security, the practitioner must demonstrate that the platform selected is secure and private, with end-to-end encryption necessary for video calls. A comprehensive risk assessment should be done on the platform; however, during the COVID-19 crisis, there was some relaxation of the need for a comprehensive assessment. Some examples of available telehealth platforms include cloud-based ones (Coviu, Doxy.me), app-based systems (Physitrack), or those that are embedded within practice software management like Cliniko [25]. Additionally, examples of commonly used public platforms include Zoom for Healthcare, which integrates some physiotherapy software and has a higher level of security with HIPAA compliance. Other platforms include Microsoft Teams, which is more secure than Skype but may have some drawbacks related to set-up difficulty, and user-friendliness [26].

Advantages of telerehab

Patients who have undergone spine surgery face accessibility concerns that have existed prior to the COVID-19 pandemic and will continue to be important factors to consider in the post-COVID era. In the past, one of the major advantages of telerehab has been its ability to circumvent physical barriers, transportation concerns, and financial limitations [27]. With stay-at-home orders issued throughout the nation, more people are discovering the convenience, efficacy, and other advantages of completing tasks remotely. Telerehab offers indirect contact between the clinician and the patient while offering the patient social interaction, access to their treating healthcare therapist, and psychological wellness, which may be interrupted as a result of physical isolation during the current circumstances [28].

Additionally, by eliminating the travel necessity between the patient and the provider, in-home rehabilitation services can decrease travel expenses and caregiver burden. This also provides convenience for patients with mobility impairments [29]. Levy et al. demonstrated this by conducting a study with 26 Veterans enrolled in the Rural Veterans Telerehab Initiative. The average length of participation for each Veteran was 99.2 days, during which the Veterans avoided an average of 2,774.7 travel miles and saved an average of $1,150.50 in travel expenses [27]. All of these factors make healthcare more accessible for spine surgery patients. Given that this modality of rehabilitation provides continuity of care from the hospital to the patient’s home, patients could be discharged earlier, which decreases the cost of healthcare [30]. In addition, with increased access to healthcare, patients will be less likely to employ inappropriate use of emergency medical services [30]. Given the advantages of telerehab in the current setting of the post-COVID era, clinicians may find more use in this modality of therapy.

Disadvantages of telerehab

As more clinicians are discovering the advantages of telerehab usage in the COVID era, the disadvantages are worth noting as well. Aside from the obvious cost and coverage concerns of telerehab, the innate lack of person-to-person contact in telerehab may make it more difficult to build a strong provider-patient relationship [29]. Physical therapy sessions in the patient’s own home is contextually different than the clinic setting. The home lacks the presence of other staff members, other patients, and other symbols that represent rehabilitation, including the therapy table [31]. Furthermore, the patient may encounter equipment barriers, including the lack of weights, bands, and medicine balls that are available in clinic settings and this may limit the amount of therapeutic exercises available for the patient [32]. Patients may also lack the technology necessary to carry out telerehab or the knowledge to use it. Adequate training, support, and information must be given to patients prior to the start of their therapy.

In addition, the lack of person-to-person contact makes it difficult for the provider to palpate and execute special tests that may be crucial for diagnostic purposes. Given the obstacles listed above, telerehab could be used as an adjunctive therapy where in-person sessions are utilized for initial evaluation and occasional follow-up appointments. It would be important to limit the possibility of overlooking red flags in patients with more complicated rehabilitation courses [33]. Table 1 summarizes the advantages and disadvantages of telerehab.

**Table 1:** Advantages and disadvantages of telerehab

Advantages	Disadvantages
Decrease travel time	Lack of equipment
Decrease travel expenses	Lack of technology
Decrease caregiver burden	Lack of person-to-person contact

Physical therapist-patient relationship

As new modalities of healthcare delivery are becoming more prevalent, it is worth exploring how the physical therapist-patient relationship evolves with telerehab. Similar to traditional face-to-face encounters, the physical therapist gives the patient one-on-one attention and personalized therapies. Hinman et al. assessed the experiences of eight physical therapists and 12 patients after physical therapy sessions over Skype [34]. The participants in this clinical trial were interviewed regarding their experiences after more than 30 physical therapy sessions each. In general, patients feel they are part of a supportive and friendly physical therapist-patient relationship [34]. With the ability to stay in their home environments, patients seem more relaxed and receptive to the therapies provided [34]. Some physical therapists feel that by virtually going into a patient’s home, the encounter is more personal than those that take place in a clinic [34]. The comfort of being in a familiar environment lays a positive foundation for building a positive therapeutic relationship [34]. The familiarity of the patients’ home environment plays a large role in perceived patient empowerment and self-management. By seeing patients in their home environment, physical therapists were able to prescribe customized therapies that were realistic and safe to do at home [34]. These therapies were tailored to tackle day-to-day obstacles that the patient may face at home, which allowed patients to feel a sense of empowerment and self-management, which is the main factor in compliance [34].

Patient satisfaction has been a priority in healthcare. The quality of patient care is measured by The Healthcare Effectiveness Data and Information Set, which measures different domains of quality, including effectiveness, access, availability, and experience of care [35]. Patient satisfaction is also associated with reimbursements from the Center for Medicare and Medicaid [35]. Overall, there have been studies that show satisfaction with telemedicine [36]. Gustke et al. evaluated patient satisfaction of 495 patient appointments and found that overall patient satisfaction was 98.3% [36]. Luptak et al. did a study on the patient satisfaction of 132 United States Veterans utilizing telehealth [37]. The study demonstrated an overall high satisfaction rating [37]. With 93 patients having used the telehealth technology for more than 10 sessions, 86 of those patients reported satisfaction or high satisfaction [37]. Despite having virtual communications, patients felt that providers adequately educated, provided access to information, and monitored progress [38]. The provider-patient relationship was not affected by this modality of healthcare delivery [38].

Usage of telerehab to date

Telerehab is generally considered new, however, its use dates back to 1976, as a telephone was utilized to treat aphasia and motor speech disorders [39]. Then, in 1987, a team led by R.T. Wertz used a closed-circuit television and a computer-controlled video laserdisc with a telephone for speech and language disorders [40]. More recently, Internet-based videoconferencing has been used to assess and treat language and motor disorders after brain impairment and Parkinson’s disease. A review of studies utilizing Lee Silverman Voice Treatment (a treatment for speech disorders associated with Parkinson's disease) demonstrated that telerehab is feasible through the combination of on-and offline assessments, online clinic visits, and online monitoring of speech or swallowing disorders [41].

Spinal cord injury patients stand to gain much from telerehab services. In 2000, Emory University conducted a study regarding patients with mobility impairment due to spinal cord injuries who were given nine weeks of video-based intervention, a telephone-based intervention, or standard follow-up care in a rehabilitation facility [42]. One year after discharge, patients who received an intervention were found to have a significantly higher Quality of Well-Being (QWB) scores, QWB of 0.53 in the intervention group as compared to 0.48 in the standard follow-up care group [42]. Depending on the type of intervention received, the mean annual hospital days varied: three days for the video group, 5.22 days for the telephone group, and 7.95 for the standard care group. This demonstrated that video and telephone interventions may improve quality of life and reduce costs due to reduced rehospitalization. In 2014, a 12-week home exercise program with supervised physical therapy via videoconference demonstrated reduced pain and improved shoulder function in manual wheelchair users with spinal cord injury and shoulder pain [43]. Table 2 summarizes the literature to date with the results and outcomes of telerehab use.

**Table 2:** Summarization of literature to date regarding the usage of telerehabilitation and outcomes

Date	Author	Title	Journal (abbreviated)	Summary
2015	Agostini, Moja, Banzi, et al. [30]	Telerehab and recovery of motor function: a systematic review and meta-analysis	J Telemed Telecare	Inconclusive evidence for the efficacy of telerehab on motor function
2015	Levy, Silverman, Jia, et al. [27]	Effects of physical therapy delivery via home video telerehab on functional and health-related quality of life outcomes	J Rehabil Res Dev	Decreased the transportation and financial burden; significant improvement between baseline and discharge; 96% of patients were satisfied or very satisfied
2016	Testa and Rossettini [31]	Enhance placebo, avoid nocebo: How contextual factors affect physiotherapy outcomes	Man Ther	Physical therapist and patient features, characteristics of the treatment, healthcare settings all influence clinical outcomes
2017	Tenforde, Hefner, Kodish-Wachs, et al. [29]	Telehealth in Physical Medicine and Rehabilitation: A Narrative Review	PM R	Limitations include barriers in establishing a strong patient relationship; limited physical examination; patients with mobility impairments benefit greatly
2018	Howard and Kaufman [32]	Telehealth applications for outpatients with neuromuscular or musculoskeletal disorders	Muscle Nerve	Promotes access to healthcare, decreases travel burden and financial burden, therefore beneficial when used to overcome physical and geographical barriers.

Increasing need in the COVID era

Social distancing, while necessary, has restricted the work of physical therapists, as they are in close contact with their patients [2]. Urgent treatments have been deemed as essential rehabilitation services by the World Confederation of Physical Therapy [44]. The suspension of non-urgent physical therapy has affected both patients, who are suffering from disabilities, and therapy professionals, who are suffering from a decrease in practice and income [45]. Telerehab offers a solution to these ailments, as it may allow access to rehabilitation care. Implementation of telerehab has been advised by national organizations such as the Chartered Society of Physiotherapy, Italian Physiotherapy Association, and American Physical Therapy [46-49]. The effects of telerehab have been investigated in various musculoskeletal (MSK) conditions, including lower back pain, lumbar stenosis, osteoarthritis, and neck pain. The benefits include reducing hospitalization rate, reducing readmission rate, shorter length of stay in the rehabilitation unit, decreased burden of access to outpatient physical therapy while improving the quality of life, health outcomes, and early return to work [4]. The low cost of Internet connection along with smart devices and applications allows telerehab to be utilized by a wide range of patients and health care professionals. The physical therapists will benefit by providing continuity of care from home [50].

## Conclusions

In conclusion, studies have shown that telerehab is well-received by patients as a stand-alone treatment or when supplemented with conventional face-to-face therapy. However, the variability in the employment of telerehab and lack of rigorous studies specifically tailored to spine patients limits the conclusion of whether telerehab services should be distributed more broadly as a mainstream rehabilitation delivery paradigm beyond the current COVID-19 pandemic landscape. Large, well-powered, long-term studies are required to definitively articulate the specific indications for telerehab in spine patients and explore the patient outcomes. It is clear that with the recent advancements in technology, and the increasing availability of low-cost platforms for telerehab services, the field will continue to expand in the future. The combination of evidence-based methodologies with cost-effective services will serve as a basis for further expansion of vital telerehab services and increase reimbursement by health insurance providers.
